# Can early cancer detection be improved in deprived areas by involving community pharmacists?

**DOI:** 10.3399/bjgp22X718865

**Published:** 2022-04-01

**Authors:** Judit Konya, Richard D Neal, Chris Clark, David Bearman, John Campbell

**Affiliations:** Exeter Collaboration for Academic Primary Care (APEx), University of Exeter, Exeter; GP Research Fellow, Kernow CIC Training Hub, Health Education England; Salaried GP, Carn to Coast Health Centres, Cornwall.; APEx, University of Exeter, Exeter.; APEx, University of Exeter, Exeter.; Local Pharmaceutical Committee, Devon.; APEx, University of Exeter, Exeter.

## INTRODUCTION

The key to the success of cancer treatments and better clinical outcomes is early detection. The incidence and mortality from cancer is higher in patients with lower socioeconomic status compared to that for more affluent patients.^1^

General practice is crucial for the early diagnosis of cancer. The COVID-19 pandemic highlighted pre-existing GP workforce and access inequalities, which are expected to get worse.^2^ The 2020 General Practice Patient Survey suggests that patients from deprived areas have more difficulties accessing general practice than patients from affluent areas.^3^ Secondary care treatment waiting times have also become longer compared to pre-pandemic levels, and have increased significantly in deprived populations.^4^

## COMMUNITY PHARMACY, HEALTH INEQUALITIES, AND CANCER

Community pharmacies are increasingly often providing consultations and advice to patients. The NHS Community Pharmacist Consultation Service was designed to relieve pressure on general practice, with patients being referred by the triaging GP surgery staff to community pharmacies for same-day consultation relating to minor illnesses.^5^

The positive pharmacy care law states that in deprived areas more people can access a community pharmacy within 20 minutes’ walk than in affluent areas.^6^ This raises the possibility that pharmacies could play a role in addressing inequalities in primary care access. Community pharmacies are contractually required to deliver up to six public health campaigns each financial year. Becoming a Healthy Living Pharmacy has been an essential service requirement since 2020/2021, which ensures that community pharmacies are consistently providing health promotion, targeting the health and wellbeing needs of their local communities, and assisting to reduce health inequalities. Both of these requirements provide opportunities to deliver cancer-related initiatives.^7^ The Royal Pharmaceutical Society has suggested that community pharmacies could become ‘early diagnosis hubs’ for cancer based on their convenience and ease of access, their potential to deliver screening and educational services, and/or to refer directly to other healthcare providers.^7^ However, in order to fulfil this potential barriers need to be overcome, including insufficient training, and uncertainty of professional status and role among policymakers and commissioners.^7^

A systematic review found that it is possible to recruit patients to community pharmacy-based programmes aimed at early cancer detection; however, there was no UK-based study included.^8^ The authors suggested that, taking the positive pharmacy care law into account, community pharmacies might contribute to reduction of health inequalities by delivering public health interventions. Their review of reviews identified only one study (out of 157), from the US, which actually explored pharmacy-based cancer screening initiatives in the context of lower socioeconomic status.^9^

## DO PATIENTS PRESENT TO COMMUNITY PHARMACISTS WITH POTENTIAL CANCER SYMPTOMS?

The Wise Up To Cancer programme was partly delivered in community pharmacies. Pharmacy Champions were trained to deliver the intervention, which consisted of a 15–30 minute assessment for members of the public, aiming to modify those behaviours associated with increased risk of developing cancer, increase public awareness of cancer signs and symptoms, participation in cancer screening, and signpost to other services. Overall, 640 of 1297 participants experienced a red flag symptom shortly prior to participation. Around two-thirds of those who were not up-to-date with screening took positive action to engage with the screening process.^10^ When repeated among South Asian women, an even higher proportion reported potential symptoms of cancer, and nearly all participants said they would be more likely to speak to their GP about their cancer signs or symptoms as a result of participating in the programme.^11^

One small qualitative study found that more than half of 25 patients diagnosed with lung, colorectal, or gastro-oesophageal cancer had initially tried to manage their red flag symptoms by purchasing over-the-counter (OTC) medication prior to diagnosis; only three participants sought help from pharmacy staff, from whom they received appropriate advice.^12^ The study identified that frequent purchases of the same medication were not explored further by staff in the community pharmacy, and participants had low awareness of the pharmacists’ healthcare advisory role. One other study focused on patients seeking advice or OTC medication for red flag symptoms. The authors reported that a higher number of red flag symptoms were presented in pharmacies in areas of high deprivation; however, the difference did not reach statistical significance and individual patients’ deprivation status was not reported.^13^

## POTENTIAL PATHWAYS

The Accelerate, Coordinate, Evaluate programme was developed by NHS England and supported by Cancer Research UK and Macmillan Cancer Support. The pharmacy/primary care cluster of the programme sought to develop the role of primary care health professionals, other than GPs, in the early diagnosis of cancer. Embedded pharmacy projects delivered awareness-raising campaigns on various cancers, provided direct access to chest X-ray, or offered a secondary care referral pathway for suspected lung cancer.^14^ The number of patients referred was 16 and 60, respectively, with patient uptake of chest X-ray or respiratory clinic referral being 62.5% and 78.0%, respectively. Although referrals from pharmacists were judged by secondary care consultants as appropriate and of high quality, no diagnosis of malignancy resulted from the referrals. Some GPs and radiologists expressed concern about the potential increase in workload, and the initiative was ultimately decommissioned on account of not achieving its objectives and associated financial concerns.

Recent qualitative exploration of a community pharmacy referral pathway for lung cancer in deprived communities found that such a pathway was both acceptable and feasible for healthcare professionals and for members of the public. There was no malignancy detected in the 12 patients who were referred for chest X-ray during the 11 month duration of the project. Barriers to uptake included low awareness of the service and the role and capacity of the pharmacists to deliver the service, while facilitators were the pharmacists’ perceived accessibility and approachability. It was highlighted that communication with the patients’ GPs was crucial.^15^

Community pharmacies can conduct questionnaire assessments and deliver interventions aiming to assess patients’ cancer risk. For example, a pharmacy-based sun-safety quiz campaign showed that patients who attended pharmacies in more deprived areas reported increased behaviours associated with a higher risk of developing skin cancer, while sun-safety knowledge scores were lower compared to patients who attended pharmacies in more affluent areas.^16^ Another study that assessed approaches to adopt a web-based cancer risk estimation tool, the Risk Estimation for Additional Cancer Testing (REACT), suggested that the best way to offer the service was to adopt a supportive approach, whereby patients complete the tool with the assistance of a healthcare professional, such as a pharmacist.^17^

## THE FUTURE

Current evidence from the UK regarding the community pharmacies’ role in early cancer diagnosis is mainly based on small scale studies with limited outcome data, and often reported in the so-called ‘grey literature’.

Community pharmacists may have an important future role in assessing symptoms of potential cancer, beyond their established educational and awareness raising role. The feasibility of various pharmacy-based approaches in the early detection of cancer has been investigated and demonstrated. Patients present to community pharmacies with potential cancer-related symptoms, observed more frequently in deprived areas,^13^ and pharmacists appear able to advise them appropriately. Direct referral pathways are feasible. There is a potential to further exploit the good access and the expertise of pharmacists in areas of deprivation; however, there are no large scale data to demonstrate improved clinical or survival outcomes. There have been no formal assessments of cost-effectiveness, and comparisons of the effectiveness of interventions between different socioeconomic groups are also needed.

An up-to-date review of the literature, including grey literature, is required, summarising recent evidence, including the details of the interventions, barriers and facilitators, and clinical outcomes. In addition, further high-quality evidence is needed in order to inform the development of new care pathways, seeking to maximise the potential for this group of primary care practitioners in improving cancer outcomes, especially in socioeconomically deprived communities and populations.
